# Polyphenols and Alzheimer's Disease: A Review on Molecular and Therapeutic Insights With In Silico Support

**DOI:** 10.1002/fsn3.70496

**Published:** 2025-08-28

**Authors:** Guifei Chen, Yan Su, Siyu Chen, Tiandong Lin, Xueying Lin

**Affiliations:** ^1^ The Second Affiliated Hospital of Hainan Medical University Haikou China; ^2^ College of Traditional Chinese Medicine Hainan Medical University Haikou China; ^3^ Hainan Hospital of Guangdong Provincial Hospital of Traditional Chinese Medicine Haikou China

**Keywords:** Alzheimer's disease, in silico approach, neuroprotection, oxidative stress, polyphenols

## Abstract

Alzheimer's disease (AD), a progressive neurodegenerative disorder, characterized by amyloid‐beta (Aβ) aggregation, tau hyperphosphorylation, oxidative stress, and neuroinflammation resulted the cognitive memory loss. Despite extensive research, effective therapeutic treatment remains elusive. Polyphenols, naturally occurring bioactive compounds, have emerged as promising neuroprotective agents due to their potent antioxidant, anti‐inflammatory, and amyloid‐modulating properties. Research indicated that these bioactive compounds have potent antioxidant and anti‐inflammatory properties, have garnered significant attention for their potential role in combating AD by targeting its key pathological mechanisms. Preclinical studies highlight the efficacy of various polyphenols, such as resveratrol, curcumin, and epigallocatechin gallate, in improving cognitive function and reducing neurodegeneration. Moreover, in silico approaches, displayed polyphenols mechanistic interactions with key AD targets to reduce pathogenesis. These computational models accelerate drug discovery by predicting binding affinities, optimizing structural modifications, and identifying novel polyphenol derivatives with enhanced therapeutic potential. This review explores the multifaceted role of polyphenols in AD mitigation, emphasizing their impact on oxidative stress and neuroinflammation while integrating in silico evidence to reinforce their therapeutic relevance. Convincingly, through the suppression of key mediators of AD, including oxidative stress, neuroinflammation, amyloid‐beta and tau proteins, polyphenols exhibited outstanding therapeutic potential to treat AD.

## Graphical abstract

Polyphenols, known for their antioxidant and anti‐inflammatory properties, show strong potential in mitigating Alzheimer's disease by targeting oxidative stress, neuroinflammation, amyloid‐beta aggregation, and tau hyperphosphorylation. Preclinical and in silico studies highlight compounds like resveratrol, curcumin, and epigallocatechin gallate for their neuroprotective effects and interaction with key Alzheimer's disease related targets. These findings underscore polyphenols as promising therapeutic agents in combating Alzheimer's disease and advancing drug discovery efforts. Represents the potential of polyphenols in mitigating pathological features of Alzheimer's Disease.

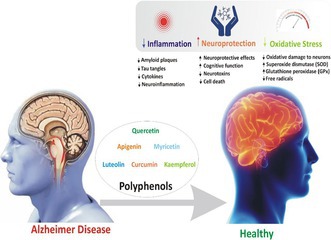

## 1 Introduction

Alzheimer's disease (AD) is a progressive neurodegenerative disorder characterized by cognitive decline, memory loss, and behavioral disturbances. It is the most common cause of dementia, affecting over 50 million people worldwide, with projections suggesting this number will triple by 2050 (As 2023; Rai et al. 2024). The pathogenesis of AD is multifactorial, involving the accumulation of amyloid‐beta (Aβ) plaques, neurofibrillary tangles (NFTs) composed of hyperphosphorylated tau protein, oxidative stress, and chronic neuroinflammation (García‐Morales et al. 2021; Simunkova et al. 2019). Despite decades of research, effective disease‐modifying therapies remain elusive, highlighting the urgent need for novel therapeutic strategies. In recent years, natural compounds, particularly polyphenols, have garnered significant attention for their potential to mitigate AD pathology through their multifaceted mechanisms of action (Liu et al. 2023).

Polyphenols are a diverse group of phytochemicals found abundantly in fruits, vegetables, tea, coffee, and wine. They are classified into flavonoids, phenolic acids, stilbenes, and lignans, each with unique structural and functional properties (Hassan et al. 2023; Pandey and Rizvi 2009). These compounds exhibit potent antioxidant, anti‐inflammatory, and neuroprotective effects, making them promising candidates for combating AD. Oxidative stress, a hallmark of AD, arises from an imbalance between reactive oxygen species (ROS) production and the brain's antioxidant defense mechanisms (Arain et al. 2018). The brain is particularly vulnerable to oxidative damage due to its high oxygen consumption, lipid‐rich content, and relatively low antioxidant capacity (Butterfield and Halliwell 2019). Polyphenols, such as curcumin, resveratrol, and epigallocatechin gallate (EGCG), have been shown to scavenge ROS, upregulate endogenous antioxidant enzymes, and chelate metal ions, thereby reducing oxidative damage to neurons (Vauzour et al. 2010; Yatao et al. 2018).

Neuroinflammation, another critical contributor to AD progression, involves the activation of microglia and astrocytes, leading to the release of pro‐inflammatory cytokines and chemokines. Chronic inflammation exacerbates neuronal damage and synaptic loss, further impairing cognitive function (Patterson 2015). Polyphenols modulate neuroinflammatory pathways by inhibiting the activation of nuclear factor‐kappa B (NF‐κB) and mitogen‐activated protein kinase (MAPK) signaling, thereby reducing the production of inflammatory mediators (Zhang et al. 2010). For instance, resveratrol has been shown to suppress microglial activation and reduce the secretion of interleukin‐1β (IL‐1β) and tumor necrosis factor‐alpha (TNF‐α) in preclinical models of AD (Capiralla et al. 2012).

In addition to their biological effects, advances in computational biology have provided valuable insights into the molecular interactions between polyphenols and AD‐related targets. In silico studies, including molecular docking and molecular dynamics simulations, have elucidated the binding affinities and mechanisms of polyphenols with key proteins such as beta‐secretase (BACE1), acetylcholinesterase (AChE), and tau (Dhakal et al. 2019). These computational approaches have not only validated the therapeutic potential of natural products including polyphenols but also guided the design of novel analogs with enhanced efficacy and bioavailability (Arain et al. 2024; Ashraf et al. 2024; Safdar et al. 2024).

Despite the promising preclinical evidence, challenges remain in translating these findings into clinical practice. Issues such as poor bioavailability, rapid metabolism, and limited blood–brain barrier permeability hinder the therapeutic application of polyphenols. However, advancements in drug delivery systems, including nanoparticles and liposomes, offer potential solutions to these limitations (Nabi, Arain, Hassan, et al. 2020; Prajapati et al. 2025; Teleanu et al. 2018). This review aims to provide a comprehensive overview of the role of polyphenols in targeting oxidative stress and neuroinflammation in AD, supported by in silico evidence, and to discuss future directions for their development as therapeutic agents.

## 2 Overview of Alzheimer's Disease

Alzheimer's disease is a progressive, irreversible neurodegenerative disorder and the most common cause of dementia, accounting for 60%–80% of all dementia cases (Alzheimer's Association 2023). It is characterized by a gradual decline in cognitive functions, including memory, reasoning, language, and the ability to perform daily activities (Rai 2021). AD typically manifests in individuals over the age of 65, although early‐onset forms of the disease can occur in younger individuals. The global prevalence of AD is staggering, with over 50 million people currently affected, and this number is projected to rise to 152 million by 2050 due to aging populations (Saxena et al. 2012).

The pathological hallmarks of AD include the accumulation of extracellular amyloid‐beta (Aβ) plaques and intracellular neurofibrillary tangles (NFTs) composed of hyperphosphorylated tau protein. Aβ plaques result from the abnormal cleavage of amyloid precursor protein (APP) by beta‐secretase (BACE1) and gamma‐secretase, leading to the aggregation of Aβ peptides, particularly Aβ42, which is highly toxic to neurons (Hardy and Selkoe 2002). NFTs, on the other hand, arise from the hyperphosphorylation of tau protein, which disrupts microtubule stability and impairs neuronal transport, ultimately leading to cell death (Iqbal et al. 2010).

In addition to these hallmark features, AD is associated with oxidative stress, neuroinflammation, synaptic dysfunction, and neuronal loss. Oxidative stress occurs due to an imbalance between the production of reactive oxygen species (ROS) and the brain's antioxidant defenses, leading to damage to lipids, proteins, and DNA (Butterfield and Halliwell 2019). Neuroinflammation, driven by the activation of microglia and astrocytes, exacerbates neuronal damage through the release of pro‐inflammatory cytokines and chemokines (Heneka et al. 2015). Synaptic dysfunction and loss, particularly in the hippocampus and cerebral cortex, are early events in AD and correlate strongly with cognitive decline (Selkoe 2002; Singh et al. 2024). The clinical progression of AD is typically divided into three stages: preclinical, mild cognitive impairment (MCI), and dementia. During the preclinical stage, pathological changes such as Aβ accumulation and tau phosphorylation occur without noticeable symptoms. In the MCI stage, individuals experience mild memory deficits but retain functional independence. As the disease progresses to the dementia stage, cognitive and functional impairments become severe, leading to complete dependence on caregivers (Jack Jr. et al. 2018).

Current treatments for AD, such as acetylcholinesterase inhibitors (e.g., donepezil) and NMDA receptor antagonists (e.g., memantine), provide symptomatic relief but do not halt or reverse disease progression. The recent approval of disease‐modifying therapies like aducanumab and lecanemab, which target Aβ plaques, has sparked hope; however, their efficacy and safety remain controversial (Cummings et al. 2021). This underscores the need for alternative therapeutic strategies that address the multifactorial nature of AD, including oxidative stress, neuroinflammation, and synaptic dysfunction. In this context, natural compounds such as polyphenols have emerged as promising candidates due to their multifaceted mechanisms of action, including antioxidant, anti‐inflammatory, and neuroprotective properties. Understanding the complex interplay of AD pathology and exploring novel therapeutic approaches are critical to developing effective treatments for this devastating disease.

## 3 Pathogenesis of Alzheimer's Disease

The pathogenesis of AD is multifactorial, involving the interplay of genetic, environmental, amyloid‐beta (Aβ) plaque deposition, tau hyperphosphorylation, oxidative stress, neuroinflammation, and synaptic dysfunction. Two hallmark pathological features of AD are the accumulation of extracellular amyloid‐beta (Aβ) plaques and intracellular neurofibrillary tangles (NFTs) composed of hyperphosphorylated tau protein (Long and Holtzman 2019). Aβ plaques result from the abnormal cleavage of amyloid precursor protein (APP) by beta‐secretase (BACE1) and gamma‐secretase, leading to the production of toxic Aβ peptides, particularly Aβ42, which aggregates and forms insoluble plaques. These plaques disrupt synaptic function, induce oxidative stress, and trigger neuroinflammation, contributing to neuronal damage and cell death (Hardy and Higgins 1992). The important pathological mechanisms contributed to the pathogenesis of Alzheimer's disease are summarized in Table 1.

**TABLE 1:** Summary of the important pathological mechanisms contributing to the pathogenesis of Alzheimer's disease.

Pathological mechanism	Description	Key effects	References
Amyloid‐beta (Aβ) Plaque Formation	Abnormal cleavage of APP by BACE1 and gamma‐secretase, leading to Aβ aggregation.	Synaptic dysfunction, oxidative stress, and neuroinflammation.	Hardy and Higgins (1992); Long and Holtzman (2019)
Tau Hyperphosphorylation	Hyperphosphorylation of tau protein leading to NFT formation.	Disruption of microtubules, impaired neuronal transport, and cell death.	Iqbal et al. (2010); Wang et al. (2019)
Oxidative Stress	Imbalance between ROS production and antioxidant defenses.	Lipid peroxidation, protein oxidation, and DNA damage.	Butterfield and Halliwell (2019); Nunomura et al. (2006)
Neuroinflammation	Activation of microglia and astrocytes, releasing pro‐inflammatory cytokines.	Neuronal damage, synaptic loss, and exacerbation of Aβ and tau pathology.	Heneka et al. (2015); Heppner et al. (2015)
Mitochondrial Dysfunction	Impaired mitochondrial function and energy metabolism.	Reduced ATP production, increased ROS, and neuronal apoptosis.	Reddy and Beal (2008); Swerdlow (2018)
Synaptic Dysfunction	Loss of synaptic connections and impaired neurotransmission.	Cognitive decline and memory impairment.	Hardy and Selkoe (2002); Terry Jr et al. (2011)
Blood–Brain Barrier (BBB) Disruption	Compromised integrity of the BBB.	Increased permeability to toxins and inflammatory cells.	Montagne et al. (2017); Sweeney et al. (2018)
Cholinergic Dysfunction	Loss of cholinergic neurons and reduced acetylcholine levels.	Impaired memory and learning.	Bartus et al. (1982); Cheng et al. (2021)
Glutamate Excitotoxicity	Excessive glutamate leading to overactivation of NMDA receptors.	Neuronal damage and cell death.	Esposito et al. (2013); Koutsilieri and Riederer (2007)
Autophagy Dysregulation	Impaired clearance of damaged organelles and protein aggregates.	Accumulation of toxic proteins and cellular debris.	Di Meco et al. (2020); Uddin et al. (2018)
Vascular Dysfunction	Reduced cerebral blood flow and vascular damage.	Hypoxia, nutrient deprivation, and Aβ accumulation.	Govindpani et al. (2019); Sweeney et al. (2019)
Epigenetic Alterations	Changes in DNA methylation and histone modification.	Altered gene expression related to AD pathology.	Sanchez‐Mut and Gräff (2015)
Protein Misfolding and Aggregation	Misfolding of proteins like Aβ and tau.	Formation of toxic oligomers and aggregates.	Ashraf et al. (2014)
Calcium Homeostasis Disruption	Dysregulation of intracellular calcium levels.	Neuronal excitotoxicity and apoptosis.	Popugaeva et al. (2017); Small (2009)
Iron Dysregulation	Accumulation of iron in the brain.	Increased oxidative stress and neuronal damage.	Mandel et al. (2007)

**FIGURE 1:**
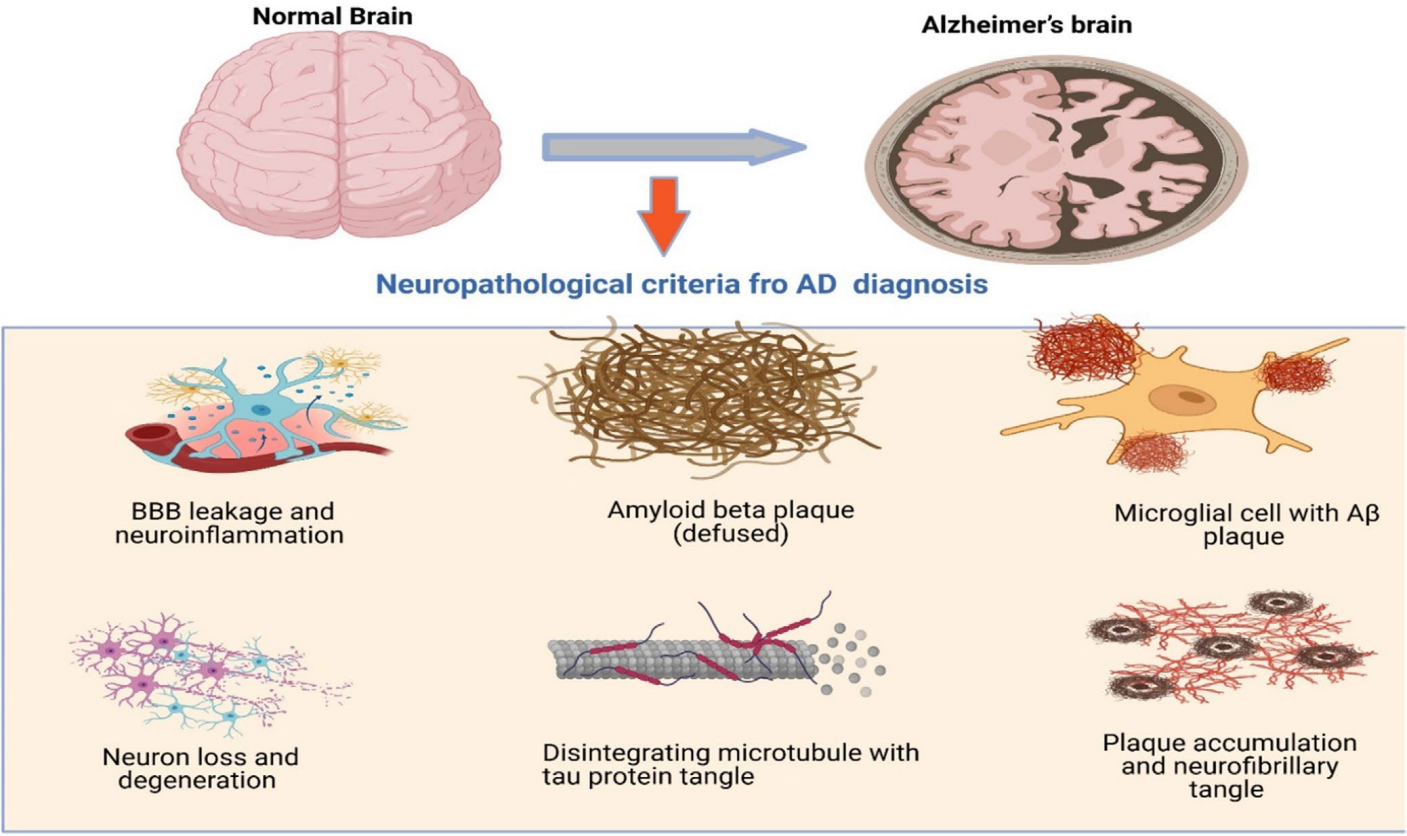
Showing the comparative neuropathological features of normal and Alzheimer's disease brain. (Created by Biorender).

NFTs, on the other hand, arise from the hyperphosphorylation of tau, a microtubule‐associated protein that stabilizes neuronal cytoskeletons. Hyperphosphorylated tau detaches from microtubules, aggregates, and forms paired helical filaments, leading to the collapse of the neuronal transport system and subsequent neurodegeneration (Han et al. 2014). Oxidative stress is another critical contributor to AD pathogenesis. The brain's high metabolic activity and lipid‐rich environment make it particularly susceptible to reactive oxygen species (ROS)‐induced damage. Oxidative stress exacerbates Aβ toxicity, promotes tau phosphorylation, and impairs mitochondrial function, further accelerating neuronal degeneration (Butterfield and Halliwell 2019). Chronic neuroinflammation, driven by the activation of microglia and astrocytes, also plays a pivotal role in AD progression. Activated glial cells release pro‐inflammatory cytokines, such as interleukin‐1β (IL‐1β) and tumor necrosis factor‐alpha (TNF‐α), which exacerbate neuronal damage and synaptic loss (Heneka et al. 2015). Additionally, genetic factors, including mutations in APP, presenilin 1 (PSEN1), and presenilin 2 (PSEN2), as well as the ε4 allele of apolipoprotein E (APOE4), increase the risk of AD by influencing amyloid and tau pathology (Karran and De Strooper 2016). Together, these interconnected mechanisms create a vicious cycle of neurodegeneration, ultimately leading to the clinical manifestations of AD. Figure 1 summarizes the pathological feature of normal and AD brain used for diagnosis.

## 4 Therapeutic Strategies for Alzheimer's Disease

Alzheimer's disease (AD) remains one of the most challenging neurodegenerative disorders to treat, with no cure currently available. However, several therapeutic strategies have been developed to manage symptoms, slow disease progression, and target underlying pathological mechanisms. These approaches can be broadly categorized into pharmacological interventions, lifestyle modifications, and emerging therapies.

### 4.1 Pharmacological Interventions for Alzheimer's Disease

Current pharmacological interventions for AD primarily focus on symptom management rather than disease modification. Acetylcholinesterase inhibitors (AChEIs), such as donepezil, rivastigmine, and galantamine, are the first‐line treatments for mild to moderate AD (Moreira et al. 2022). These drugs enhance cholinergic neurotransmission by inhibiting the breakdown of acetylcholine, thereby improving cognitive and functional symptoms (Cummings et al. 2019). Memantine, an NMDA receptor antagonist, is approved for moderate to severe AD. It regulates glutamate activity, preventing excitotoxicity and slowing disease progression (McShane et al. 1996). Recently, aducanumab, a monoclonal antibody targeting amyloid‐beta (Aβ), received conditional approval as the first disease‐modifying therapy for AD, though its efficacy remains controversial (Knopman et al. 2021). Ongoing research aims to develop therapies targeting tau pathology, neuroinflammation, and oxidative stress to address the underlying mechanisms of AD.

### 4.2 Lifestyle and Non‐Pharmacological Interventions for Alzheimer's Disease

Lifestyle modifications and non‐pharmacological interventions play a crucial role in managing AD and improving quality of life. Regular physical exercise, such as aerobic and resistance training, has been shown to enhance cognitive function, reduce neuroinflammation, and promote neurogenesis (Ngandu et al. 2015). A balanced diet, particularly the Mediterranean or MIND diet, rich in fruits, vegetables, whole grains, and omega‐3 fatty acids, may slow cognitive decline by reducing oxidative stress and inflammation (Arain et al. 2023; Morris et al. 2015). Cognitive stimulation through puzzles, reading, and social engagement helps maintain mental agility and delay symptom progression. Additionally, mindfulness‐based stress reduction and adequate sleep hygiene can mitigate stress and improve overall brain health. These interventions, when combined, offer a holistic approach to managing AD.

### 4.3 Emerging and Combination Therapies for Alzheimer's Disease

Emerging therapies for AD, targeted the key pathological mechanisms, including amyloid‐beta (Aβ) plaques, tau tangles, oxidative stress, and neuroinflammation. Monoclonal antibodies like lecanemab and donanemab have demonstrated efficacy in clinical trials by clearing Aβ plaques and slowing cognitive decline (Swanson et al. 2021). Tau‐focused therapies, such as aggregation inhibitors and anti‐tau antibodies, aim to prevent neurofibrillary tangle formation (Zempel et al. 2013). Additionally, natural compounds like polyphenols are being investigated for their antioxidant and anti‐inflammatory effects, while gene‐editing technologies, including CRISPR, offer potential for modulating AD‐related genes (Ullah et al. 2024). These approaches, combined with advanced drug delivery systems, represent promising avenues for disease‐modifying treatments.

Combination therapies address AD's multifactorial nature by simultaneously targeting Aβ, tau, oxidative stress, and inflammation. For instance, anti‐amyloid antibodies like aducanumab are being paired with tau inhibitors and anti‐inflammatory agents to enhance therapeutic outcomes (Cummings et al. 2021). Polyphenols are also explored as adjuncts to conventional therapies due to their neuroprotective properties (Kumari et al. 2022). While combination strategies hold promise for slowing disease progression, challenges such as drug interactions and optimal dosing require further research to maximize efficacy and safety.

## 5 Therapeutic Properties of Polyphenols

Polyphenols are bioactive compounds found in plant‐based foods like fruits, vegetables, tea, and wine, known for their diverse therapeutic properties. They are classified into flavonoids, phenolic acids, stilbenes, and lignans, each contributing to their health benefits (Alagawany et al. 2020; Rehman et al. 2025). Polyphenols exhibit potent antioxidant activity by scavenging reactive oxygen species (ROS) and enhancing endogenous antioxidant defenses, protecting cells from oxidative damage linked to chronic diseases such as Alzheimer's disease (AD) and cardiovascular disorders (Nabi, Arain, Rajput, et al. 2020; Pandey and Rizvi 2009). Their anti‐inflammatory effects are mediated through the inhibition of pro‐inflammatory pathways like NF‐κB and MAPK, reducing the production of cytokines such as IL‐1β and TNF‐α (Buzdar et al. 2025; Zhang et al. 2010).

Polyphenols also show neuroprotective properties by inhibiting amyloid‐beta (Aβ) aggregation and tau hyperphosphorylation, key features of AD (Freyssin et al. 2018). Additionally, they exhibit cardioprotective, anticancer, and antidiabetic effects by improving endothelial function, modulating cell signaling pathways, and enhancing insulin sensitivity (Qian et al. 2022). Despite their potential, challenges like low bioavailability require innovative delivery systems for clinical application.

### 5.1 Polyphenols and Oxidative Stress

Polyphenols are renowned for their potent antioxidant properties, which play a critical role in mitigating oxidative stress, a key contributor to the pathogenesis of numerous chronic diseases, including neurodegenerative disorders, cardiovascular diseases, and cancer (Saeed et al. 2021; Saeed, Naveed, et al. 2017b). Oxidative stress arises from an imbalance between the production of reactive oxygen species (ROS) and the body's antioxidant defense mechanisms. The brain is particularly vulnerable to oxidative damage due to its high oxygen consumption, lipid‐rich environment, and relatively low antioxidant capacity (Butterfield and Halliwell 2019). Polyphenols, such as flavonoids, phenolic acids, and stilbenes, exhibit multifaceted mechanisms in mitigating oxidative stress, particularly in the context of AD, where oxidative imbalance significantly contributes to disease onset and progression.

Polyphenols directly scavenge ROS, including superoxide anions, hydroxyl radicals, and peroxynitrite, remain fundamental (Pandey and Rizvi 2009; Saeed, Babazadeh, et al. 2017a), emerging evidence highlights the pivotal role of polyphenols in modulating intracellular signaling cascades associated with oxidative stress and neuroinflammation. Additionally, polyphenols chelate metal ions like iron and copper, which catalyze the formation of highly reactive hydroxyl radicals through Fenton reactions, further reducing oxidative damage (Fraga et al. 2010). Notably, several polyphenols exert neuroprotection by influencing the nuclear factor erythroid 2‐related factor 2 (Nrf2), nuclear factor‐kappa B (NF‐κB), and mitogen‐activated protein kinase (MAPK) pathways, which are intricately involved in redox homeostasis, inflammatory responses, and neuronal survival (Jazvinšćak Jembrek et al. 2021; Moratilla‐Rivera et al. 2023). Epigallocatechin gallate (EGCG) for instance, not only enhances endogenous antioxidant enzyme activities such as superoxide dismutase, catalase, and glutathione peroxidase but also promotes Nrf2 nuclear translocation, leading to the transcriptional activation of antioxidant response element (ARE)‐driven genes (Tang et al. 2024). Recently, (Tang et al. 2024) demonstrated that administering EGCG at a dose of 50 mg/kg significantly reduced oxidative stress in Alzheimer's disease models by activating the Keap1/Nrf2 signaling pathway. This activation increased the expression of critical antioxidant enzymes, including HO‐1 and NQO1, resulting in enhanced neuroprotection and improved cognitive function. Furthermore, in vitro findings corroborate these effects, revealing that EGCG at an effective concentration of 10 μM significantly mitigates β‐amyloid‐induced oxidative and nitrosative stress. Specifically, EGCG elevated the activities of antioxidant enzymes such as SOD and CAT, while concurrently reducing the levels of ROS and reactive nitrogen species (RNS), thereby preventing neuronal apoptosis (Kim et al. 2009). Similarly, resveratrol attenuates NF‐κB‐mediated neuroinflammatory signaling, thereby reducing the expression of pro‐inflammatory cytokines like TNF‐α and IL‐1β, which are elevated in AD brains (Singh et al. 2020). Quercetin, another potent flavonoid, demonstrates dual modulation by both activating Nrf2 and inhibiting MAPK signaling cascades, thereby suppressing oxidative stressinduced neuronal apoptosis (Islam et al. 2025; Saeed, Naveed, et al. 2017b).

Comparative analyses of these polyphenols across different in vivo and in vitro AD models reveal differential efficacy; for example, EGCG has shown pronounced inhibition of amyloid‐beta aggregation and ROS generation in transgenic mouse models, whereas curcumin primarily targets tau phosphorylation and microglial activation via downregulation of NF‐κB and MAPK pathways (Henríquez et al. 2020; Wu et al. 2022). Such mechanistic distinctions underscore the need for a hierarchical evaluation of polyphenolic efficacy tailored to specific pathological features of AD. While the antioxidant and metal‐chelating capabilities of polyphenols contribute to general neuroprotection, their signaling‐specific actions determine their therapeutic precision. Overall, a deeper mechanistic understanding reveals that polyphenols are not only act as ROS scavengers but also serve as dynamic modulators of intracellular networks, offering a promising, nuanced approach to counteracting oxidative stress and neuroinflammation in AD pathogenesis.

### 5.2 Polyphenols and Inflammation

Polyphenols, a diverse group of plant‐derived bioactive compounds, effectively modulate inflammatory pathways and reduce the production of pro‐inflammatory mediators, making them valuable in managing chronic inflammatory diseases, including neurodegenerative disorders, cardiovascular diseases, and metabolic syndromes. Mechanistically, polyphenols exert their anti‐inflammatory and neuroprotective effects primarily through the modulation of the key intracellular signaling pathways, such as NF‐κB, Nrf2, and MAPK (Du et al. 2024). These pathways orchestrate the cellular response to oxidative insults and inflammatory stimuli, leading to the regulation of the expression of inflammatory cytokines, chemokines, and enzymes like cyclooxygenase‐2 (COX‐2) and inducible nitric oxide synthase (iNOS) (Zhang et al. 2010). For instance, curcumin inhibits NF‐κB signaling by preventing IκB degradation and subsequent nuclear translocation of NF‐κB subunits, thereby suppressing the expression of pro‐inflammatory cytokines such as TNF‐α, IL‐1β, and IL‐6 (Fuloria et al. 2022). Concurrently, it activates the Nrf2 pathway, promoting the transcription of antioxidant response element (ARE)‐dependent genes like heme oxygenase‐1 (HO‐1) and NAD(P)H quinone dehydrogenase 1 (NQO1), which enhance cellular antioxidant defenses (Balogun et al. 2003). Resveratrol, another potent polyphenol, modulates both MAPK and NF‐κB signaling in AD models, where it attenuates microglial activation, reduces pro‐inflammatory cytokine release, and protects against amyloid‐beta (Aβ)‐induced neurotoxicity (Capiralla et al. 2012). Furthermore, EGCG derived from green tea similarly suppresses MAPK signaling and promotes Nrf2‐mediated antioxidant gene expression, reducing oxidative damage and inflammation in neuronal cells (Valverde‐Salazar et al. 2023).

Beyond pathway inhibition, polyphenols modulate immune responses by influencing macrophage and microglial polarization, shifting from a pro‐inflammatory M1 phenotype to an anti‐inflammatory M2 state, thereby enhancing the release of anti‐inflammatory mediators such as IL‐10 and arginase‐1 (Di Meo et al. 2020). Among various polyphenols, resveratrol has emerged as a particularly promising neuroprotective agent against AD (Pasinetti 2012). It was reported that resveratrol modulates several critical aspects of AD pathology, including the aggregation of beta‐amyloid peptides, oxidative stress, and chronic neuroinflammation (Pasinetti 2012). Preclinical studies have demonstrated that prolonged administration of resveratrol at doses of 100 mg/kg/day significantly attenuates AD‐like neuropathological changes in animal models (Pasinetti 2012). These protective effects are attributed to resveratrol's pleiotropic mechanisms of action, which include its potent antioxidant capacity, suppression of pro‐inflammatory signaling pathways such as NF‐κB, and activation of sirtuin‐1 (SIRT1) a longevity‐associated deacetylase implicated in neuronal survival and metabolic regulation. Comparative analyses across various AD models suggest curcumin and resveratrol are among the most effective polyphenols in attenuating neuroinflammation and cognitive decline, although EGCG shows promising results in reducing Aβ aggregation and oxidative stress. These differences in efficacy may reflect variances in bioavailability, blood–brain barrier permeability, and specificity toward signaling cascades. For example, while curcumin has strong NF‐κB and Nrf2 modulation capabilities, its limited bioavailability necessitates formulation enhancements for clinical application (McGrattan et al. 2019). In contrast, resveratrol demonstrates better brain penetration but may require higher doses to achieve therapeutic effects (Andrade et al. 2018). Collectively, polyphenols represent a multifaceted therapeutic strategy against AD by simultaneously modulating oxidative and inflammatory signaling pathways, with their comparative efficacy contingent upon molecular targets, pharmacokinetics, and model‐specific pathophysiology.

### 5.3 Polyphenols and Neuroprotection

Polyphenols play a significant role in neuroprotection by targeting key mechanisms implicated in neurodegenerative diseases like AD, Parkinson's disease (PD), and other cognitive disorders. Their role in neuroprotection is primarily attributed to their ability to mitigate oxidative stress, reduce neuroinflammation, modulate neurotransmitter activity, and enhance neurogenesis (Butterfield and Halliwell 2019). Their therapeutic effects are mediated through modulation of redox‐sensitive signaling pathways, suppression of pro‐inflammatory cytokines (IL‐1β, TNF‐α) via NF‐κB and MAPK inhibition, and prevention of amyloid‐beta (Aβ) and tau protein aggregation (Fernandes et al. 2021; Yahfoufi et al. 2018). One of the critical advantages of polyphenols is their capacity to cross the blood–brain barrier, allowing them to exert direct antioxidant and anti‐inflammatory actions within neural tissues. Previously, (Figueira, Garcia, et al. 2017b) demonstrated that specific polyphenol metabolites, such as catechin derivatives and phenyl‐γ‐valerolactones, selectively accumulate in brain endothelial cells and neurons, where they mitigate oxidative damage.

Recent advancements in nanotechnology have further enhanced the bioavailability and therapeutic efficacy of polyphenols. For example, (Mamashli et al. 2023) demonstrated that propolis‐derived polyphenol nanosheets exhibit robust neuroprotective effects in rotenone‐induced PD models. These nanosheets significantly improved neuronal survival, reduced oxidative damage, preserved dopaminergic neurons, and inhibited α‐synuclein aggregation (Mamashli et al. 2023). Notably, the optimal in vivo neuroprotective dose was identified as 0.5 mg/kg, which produced substantial behavioral and histological improvements. In a randomized, double‐blind, placebo controlled clinical trial, (Carrillo et al. 2025) evaluated the cognitive and neuroprotective effects of a polyphenol rich nutraceutical, Juice Plus + Premium. The supplement, which contains over 119 distinct polyphenolic compounds derived from fruit, vegetable, and berry juice powders, was administered daily over two 16‐week periods with a 4‐week washout. The trial, involving 92 participants, reported improvements in cognitive function and neuroprotective biomarkers, underscoring the clinical relevance of dietary polyphenol supplementation (Carrillo et al. 2025). Curcumin, a well‐studied polyphenol with potent anti‐oxidative properties, has shown significant neuroprotective effects in preclinical studies (Figueira, Garcia, et al. 2017b). It was reported that curcumin administration at 100 mg/kg body weight/day for 14–21 days significantly improved spatial memory, reduced lipid peroxidation, and restored the activities of key antioxidant enzymes, including SOD, CAT, and GPx, in rat models of homocysteine induced neurotoxicity (Ataie et al. 2010; Figueira, Garcia, et al. 2017b). Moreover, (Rajasekar et al. 2013) demonstrated that curcumin attenuates okadaic acid induced memory impairment in mice. At the same effective dose, curcumin improved cognitive performance and reduced markers of oxidative stress and neuroinflammation, highlighting its therapeutic promise in tauopathy associated neurodegeneration (Rajasekar et al. 2013).

Despite promising preclinical outcomes, the translational potential of polyphenols into clinical settings remains challenged by issues of low bioavailability, limited blood brain barrier (BBB) permeability, and metabolic instability factors—critical for their efficacy in human neurodegenerative conditions. Most polyphenols undergo extensive first pass metabolism and conjugation, significantly reducing their bioactive concentrations in the brain (Zhang et al. 2022). Moreover, the ability of polyphenols to penetrate the BBB is compound specific and often suboptimal, necessitating advanced delivery strategies such as nanoformulations or prodrugs to enhance CNS targeting (Ramakrishna et al. 2025; Samal et al. 2025). Collectively, these findings underscore the multifaceted neuroprotective potential of polyphenols through antioxidant, anti‐inflammatory, anti‐amyloidogenic, and neurogenic mechanisms. Their favorable pharmacokinetics, particularly BBB permeability, along with emerging nanotechnological strategies, further strengthen their candidacy as therapeutic agents against neurodegenerative diseases.

### 5.4 Polyphenols and Alzheimer's Disease

Recently polyphenols, have gained significant attention for their potential therapeutic option against AD. Polyphenols, inhibit amyloid‐beta (Aβ) aggregation and tau hyperphosphorylation, hallmarks of AD pathology (Kabir et al. 2023), while modulating neuroinflammation by attenuating microglial and astrocytic activation (Tayab et al. 2022). Beyond these cellular mechanisms, polyphenols promote synaptic plasticity, stimulate neurogenesis, and support mitochondrial function, thereby improving neuronal resilience and cognitive function (Spencer 2020). Despite promising preclinical data, the translational potential of polyphenols to human clinical settings remains constrained by their poor bioavailability, limited blood–brain barrier (BBB) penetration, and rapid metabolic degradation (Figueira, Menezes, et al. 2017a). Moreover, only select polyphenols or their derivatives demonstrate the physicochemical properties necessary to traverse the BBB effectively, a prerequisite for central nervous system activity (Velásquez‐Jiménez et al. 2021). Addressing these limitations through novel drug delivery systems such as nanoformulations or structural modifications is crucial for enhancing their pharmacokinetic and pharmacodynamic profiles in humans. The key polyphenols having Anti‐Alzheimer's properties are depicted in Table 2.

**TABLE 2:** Summary of important polyphenols with anti‐Alzheimer's properties, highlighting their sources, mechanisms, and key findings.

Polyphenol	Source	Mechanism of action	Key findings	References
Resveratrol	Grapes, red wine	Inhibits Aβ aggregation, reduces oxidative stress, anti‐inflammatory	Reduced Aβ plaques, improved cognitive function in AD models	Wang et al. (2018)
Curcumin	Turmeric	Anti‐inflammatory, antioxidant, inhibits Aβ and tau aggregation	Reduced Aβ plaques and tau tangles, improved memory in animal models	Liu et al. (2020)
EGCG	Green tea	Inhibits tau fibrillization, antioxidant, reduces Aβ toxicity	Prevented tau aggregation, reduced oxidative stress in AD models	Mandel et al. (2011)
Quercetin	Apples, onions	Antioxidant, anti‐inflammatory, inhibits Aβ aggregation	Reduced Aβ‐induced toxicity, improved synaptic plasticity	Sabogal‐Guáqueta et al. (2015)
Catechins	Green tea	Antioxidant, anti‐inflammatory, inhibits Aβ and tau aggregation	Reduced Aβ plaques, improved cognitive function in AD models	Weinreb et al. (2009)
Ferulic Acid	Whole grains, coffee	Antioxidant, anti‐inflammatory, inhibits Aβ aggregation	Reduced oxidative stress and Aβ plaques, improved memory in AD mice	Yan et al. (2023)
Apigenin	Parsley, celery	Anti‐inflammatory, antioxidant, promotes neurogenesis	Reduced neuroinflammation, enhanced cognitive function in AD models	Zhao, Wang, Liu, et al. (2013)
Luteolin	Celery, peppers	Antioxidant, anti‐inflammatory, inhibits Aβ aggregation	Reduced Aβ‐induced toxicity, improved synaptic plasticity	Jang et al. (2010)
Genistein	Soybeans	Antioxidant, anti‐inflammatory, inhibits Aβ aggregation	Reduced Aβ plaques, improved memory in AD mice	Bagheri et al. (2011)
Kaempferol	Broccoli, tea	Antioxidant, anti‐inflammatory, inhibits Aβ aggregation	Reduced oxidative stress and Aβ toxicity, improved cognitive function	Chen et al. (2018)
Oleuropein	Olive oil	Antioxidant, anti‐inflammatory, inhibits Aβ aggregation	Reduced Aβ plaques, improved memory in AD models	Luccarini et al. (2015)
Pterostilbene	Blueberries	Antioxidant, anti‐inflammatory, inhibits Aβ aggregation	Reduced Aβ plaques, improved cognitive function in AD models	Chang et al. (2012)
Anthocyanins	Berries	Antioxidant, anti‐inflammatory, inhibits Aβ aggregation	Reduced oxidative stress and Aβ toxicity, improved memory in AD models	Shih et al. (2011)
Rosmarinic Acid	Rosemary, sage	Antioxidant, anti‐inflammatory, inhibits Aβ aggregation	Reduced Aβ plaques, improved cognitive function in AD models	Habtemariam (2016)
Fisetin	Strawberries, apples	Antioxidant, anti‐inflammatory, promotes neurogenesis	Reduced Aβ‐induced toxicity, improved synaptic plasticity	Maher (2009)

### 5.5 Anti‐Alzheimer Potential of Resveratrol

Resveratrol, a naturally occurring polyphenol found in grapes, red wine, and berries, has garnered significant attention in targeting AD through multiple mechanisms, including inhibition of Aβ aggregation, attenuation of tau pathology, suppression of oxidative stress, and reduction of neuroinflammation. It facilitate Aβ clearance by upregulating neprilysin expression and activating autophagy, while also inhibiting tau hyperphosphorylation via modulation of kinases such as GSK‐3β (El‐Sayed and Bayan 2015; Sánchez‐Melgar et al. 2022). Furthermore, resveratrol exhibits potent antioxidant activity by scavenging reactive oxygen species (ROS), enhancing endogenous antioxidant defenses, and chelating neurotoxic metal ions, thereby preserving neuronal integrity (Arbo et al. 2020). Its anti‐inflammatory effects are mediated through suppression of microglial and astrocytic activation, reduced release of pro‐inflammatory cytokines such as IL‐1β and TNF‐α, and inhibition of NF‐κB signaling, all of which help mitigate the chronic neuroinflammation implicated in AD progression (Capiralla et al. 2012).

**FIGURE 2:**
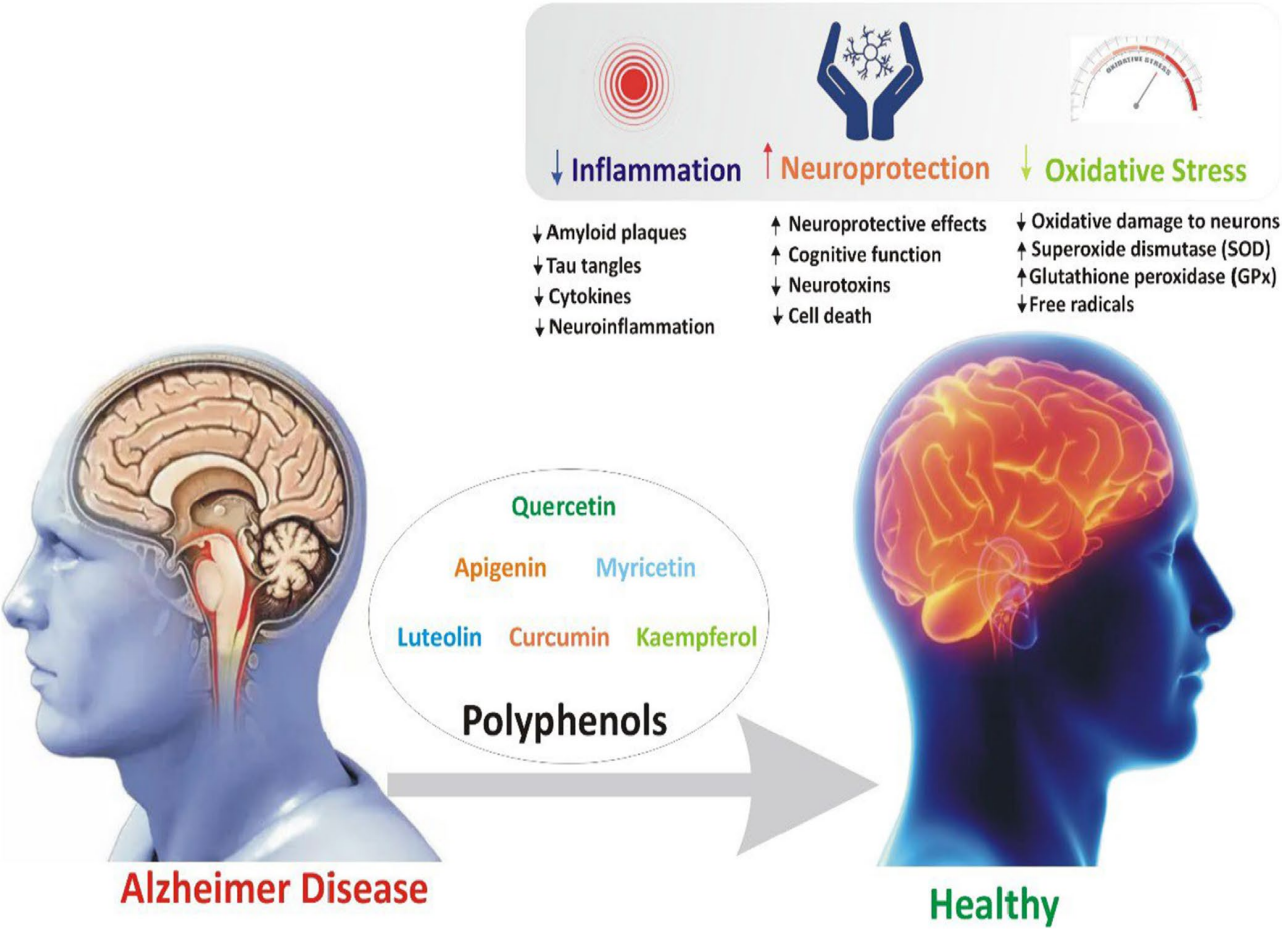
Represents the potential of polyphenols in mitigating pathological features of Alzheimer's disease.

In addition to neuroprotection, resveratrol supports synaptic plasticity, mitochondrial function, and neurogenesis, contributing to cognitive enhancement in animal models (Moussa et al. 2017). Furthermore, (Zhang et al. 2023) highlighted the therapeutic potential of three natural compounds—hamamelitannin, flavokawain A, and triacetyl resveratrol demonstrating—their dual anti‐colon cancer and neuroprotective activities through molecular docking studies, which revealed strong binding affinities to critical targets like BACE1 and CDK8. Complementing these findings, (Pan et al. 2014) reported that resveratrol derivatives possessed multi‐target neuroprotective activities, including potent inhibition of AChE and BuChE (IC_50_ as low as 0.32 μM), suppression of Aβ_1–42_ aggregation, and monoamine oxidase inhibition (IC_50_ down to 1.75 μM), significantly outperforming native resveratrol and suggesting enhanced efficacy against Alzheimer's pathology. Similarly, (Ozpak and Bağca 2024) demonstrated that resveratrol modulates the PI3K/Akt/GSK‐3β signaling pathway and reduces the expression of MMP‐2 and MMP‐9, key mediators of neuroinflammation thereby attenuating neuronal damage and reinforcing its neurotherapeutic potential. Further expanding on resveratrol's scaffold, (Tang et al. 2019) synthesized isoprenylated dimer derivatives, identifying compound 6c as a particularly promising candidate due to its strong inhibition of Aβ42 aggregation (IC_50_ = 7.56 μM), AChE activity (IC_50_ = 0.69 μM), and oxidative stress protection in SH‐SY5Y cells, along with confirmed blood–brain barrier permeability. Collectively, these findings emphasize the multifunctional therapeutic potential of resveratrol and its derivatives in targeting key pathways involved in neurodegenerative diseases, supporting a multi‐target drug development approach. Figure 2 shows the potential polyphenols combat via suppressing the pathological hallmarks of AD.

### 5.6 Anti‐Alzheimer Potential of Curcumin

Curcumin, a polyphenolic compound derived from turmeric ( *Curcuma longa* ), demonstrates significant anti‐Alzheimer's potential through its multi‐targeted actions on hallmark pathological features of AD. Curcumin effectively scavenges ROS and enhances endogenous antioxidant enzymes, thereby mitigating oxidative stress, a key driver of neuronal damage in AD (Butterfield et al. 2023). It also inhibits the formation and aggregation of Aβ plaques by binding to Aβ peptides and promoting their clearance, as demonstrated in preclinical studies (Lv et al. 2023). Furthermore, it attenuates tau hyperphosphorylation, preventing neurofibrillary tangle formation and associated neurodegeneration (Zempel et al. 2013). Its anti‐inflammatory properties suppress microglial and astrocyte activation, inhibit NF‐κB signaling, and lower pro‐inflammatory cytokines such as IL‐1β and TNF‐α, thereby alleviating chronic neuroinflammation that exacerbates AD pathology (McGrattan et al. 2019). Additionally, curcumin promotes synaptic plasticity, neurogenesis, and mitochondrial function, supporting cognitive health (Spencer 2020).

Advances in curcumin derivatives, such as steroidal and dimethylaminomethyl‐substituted analogues, have enhanced neuroprotective efficacy by improving bioavailability and multi‐target engagement, demonstrating dose‐dependent cognitive improvements and inhibition of acetylcholinesterase and Aβ aggregation in animal models (Elmegeed et al. 2015; Fang et al. 2014). Synergistic effects with probiotics like *Lactobacillus rhamnosus* further potentiate curcumin's benefits in memory and oxidative stress reduction (Patel et al. 2020). Moreover, novel bivalent and hybrid curcumin compounds show superior membrane integration and multi‐pathway inhibition, enhancing their therapeutic promise (Ausili et al. 2021; Chainoglou et al. 2020). Additionally, nanotechnology based delivery systems, such as curcumin‐loaded lipid nanocarriers, significantly improve brain targeting and efficacy, reducing Aβ pathology, oxidative stress, and neuroinflammation more effectively than free curcumin (Far et al. 2024). Collectively, these research advancements underscore curcumin's robust neuroprotective profile and its potential as a natural, multi‐faceted therapeutic agent for AD, with ongoing clinical studies focused on optimizing delivery and dosing strategies to overcome bioavailability challenges and maximize clinical benefits.

### 5.7 Anti‐Alzheimer Potential of Quercetin

Quercetin, a naturally abundant flavonoid found in apples, berries, onions, and tea, has shown significant promise for its multifaceted neuroprotective effects against AD. Its potent antioxidant activity helps to neutralize the ROS and enhance endogenous antioxidant defenses, reducing oxidative damage to neurons, a key driver of AD progression (Butterfield and Halliwell 2019). It was reported that quercetin also inhibits the formation and aggregation of Aβ plaques by modulating the cleavage of amyloid precursor protein (APP) and promoting Aβ clearance (Ansari et al. 2009). Additionally, it attenuates tau hyperphosphorylation, preventing the formation of neurofibrillary tangles, another hallmark of AD pathology. Its anti‐inflammatory effects are mediated through the suppression of NF‐κB and MAPK signaling pathways, leading to reduced production of pro‐inflammatory cytokines such as IL‐1β and TNF‐α, and inhibition of microglial activation (Chiang et al. 2023). Beyond these actions, quercetin improves synaptic plasticity, promotes neurogenesis, and improves mitochondrial function, collectively enhancing cognitive health and neuronal survival (Grewal et al. 2021). Preclinical studies across diverse models, including zebrafish and rodent systems, consistently demonstrate quercetin's efficacy in improving memory, learning, and behavioral outcomes (Jangdey et al. 2018; Mani et al. 2018). Recent advancements in quercetin derivatives, such as quercetin‐1,2,3‐triazole hybrids, have shown enhanced cholinesterase inhibition and antioxidant activity, surpassing standard drugs like galantamine, thus highlighting quercetin's potential in multitarget AD therapy (Carreiro et al. 2023). Additionally, quercetin's modulation of neuronal microRNAs and downregulation of key enzymes like BACE1 further underscores its molecular‐level benefits in reducing amyloidogenesis and improving neuronal function (Al‐Sawasany et al. 2025; Qi et al. 2020). Collectively, these findings position quercetin as a promising natural compound for both preventive and therapeutic strategies against AD, acting through antioxidative, anti‐amyloidogenic, anti‐inflammatory, and neurogenic mechanisms.

### 5.8 Anti‐Alzheimer Potential of Kaempferol

Kaempferol, a natural flavonoid found in foods like tea, broccoli, and apples, has shown significant promise in combating AD. Kaempferol mitigates AD pathology in patients via potent antioxidant, anti‐inflammatory, and anti‐amyloidogenic actions (M Calderón‐Montaño et al. 2011). It also inhibits the aggregation of amyloid‐beta (Aβ) peptides and hyperphosphorylated tau protein—hallmarks of AD—by modulating kinases like GSK‐3β, thus preventing plaque and tangle formation (Rahul and Siddique 2021). Its anti‐inflammatory efficacy is evidenced by the suppression of microglial and astrocytic activation and downregulation of pro‐inflammatory cytokines such as IL‐1β and TNF‐α via NF‐κB pathway inhibition (Chen et al. 2022). Beyond these, kaempferol promotes synaptic plasticity, neurogenesis, and mitochondrial function, enhancing cognitive performance (Alrumaihi et al. 2024).

Studies in rodents and Drosophila models have confirmed its ability to reverse memory deficits and reduce neurodegeneration at doses ranging from 10 to 50 mg/kg or 10 μM (Babaei et al. 2021; Dong et al. 2023; Kim et al. 2010). Importantly, its therapeutic efficacy is amplified when co‐administered with borneol, which enhances kaempferol's blood brain barrier permeability and central nervous system bioavailability (Zhang et al. 2015). Polyherbal formulations containing kaempferol and quercetin have also shown synergistic effects in reducing oxidative stress, neuroinflammation, and cognitive decline in AD models (Alexander et al. 2023). Moreover, kaempferol enriched plant extracts exhibit significant cholinesterase inhibition, highlighting their potential in symptomatic management of AD (Chukwuma et al. 2023). Collectively, these findings substantiate kaempferol's therapeutic promise as a safe, multifactorial candidate for both prevention and treatment of Alzheimer's disease by targeting oxidative stress, neuroinflammation, amyloid pathology, and neuronal dysfunction.

### 5.9 Anti‐Alzheimer Potential of Naringenin

Naringenin, a citrus derived bioactive flavonoid, has emerged as a promising neurotherapeutic candidate for AD, owing to its multifaceted mechanisms targeting key pathological features against pathological disorder. Extensive preclinical research has demonstrated that naringenin modulates critical pathways involved in AD progression, including amyloid‐β (Aβ) deposition, tau hyperphosphorylation, oxidative stress, neuroinflammation, and synaptic dysfunction. It exhibits potent antioxidant properties by enhancing endogenous defense systems, thereby mitigating oxidative neuronal damage a central event in AD pathogenesis (Heo et al. 2004). Furthermore, naringenin interferes with Aβ plaque formation by regulating amyloid precursor protein (APP) processing, reducing Aβ production, and preventing tau hyperphosphorylation, thereby safeguarding neurons from aggregation‐induced toxicity (D'Egidio et al. 2025). In parallel, it exhibits anti‐inflammatory actions by suppressing microglial activation and downregulating pro‐inflammatory cytokines such as IL‐1β and TNF‐α, attenuating the chronic neuroinflammation implicated in neuronal degeneration (Yang et al. 2014). Beyond its anti‐oxidative and anti‐inflammatory effects, naringenin promotes synaptic plasticity and neurogenesis, essential for maintaining cognitive performance, and supports mitochondrial function, which is often compromised in AD, thereby alleviating neuronal energy deficits (Davinelli et al. 2023).

Multiple in vivo studies corroborate naringenin's therapeutic efficacy in AD animal models. For instance (Ghofrani et al. 2015) reported significant improvements in learning and memory in rats administered 50 mg/kg/day of naringenin, highlighting its modulation of neuroprotective signaling pathways. Similarly, (Khan et al. 2012) demonstrated that oral administration of naringenin at 50 mg/kg body weight improved cognitive performance, reduced oxidative markers, and alleviated neuroinflammation in intracerebroventricular streptozotocin (ICV‐STZ)‐induced AD models (Ma, Yang, et al. 2013a). Furthermore, further confirmed these neuroprotective effects by showing reduced Aβ deposition and neuroinflammation following naringenin treatment (Zhu et al. 2024). Additionally, contemporary studies demonstrated the role of naringin in modulating neurodegenerative pathways by improving mitochondrial integrity and reducing neuroinflammatory markers, thereby enhancing memory and neuronal survival (Meng et al. 2021; Sachdeva et al. 2014).

Advanced pharmacological approaches have further elevated the therapeutic promise of naringenin. It was reported that well‐designed biomimetic nano‐drug delivery system encapsulating naringeninsignificantly enhanced blood–brain barrier permeability and neuronal uptake (Yang et al. 2014). However, the reduction of the administrative dose (20 mg/kg) of this nanoformulation alleviated cognitive deficits, suppressed Aβ pathology, and reduced neuroinflammation more efficiently than conventional delivery methods (Yang et al. 2014). Similarly, (Wu et al. 2021) synthesized novel naringenin carbamate derivatives, with compound 5 g exhibiting potent acetylcholinesterase (AChE) inhibition (IC_50_ = 0.42 μM), antioxidant activity, and metal chelating properties, leading to the mitigation of the adverse effects of AD pathology. Furthermore, computational studies revealed that naringenin showed high binding affinity (9.5 kcal/mol) for collapsin response mediator protein‐2 (CRMP‐2), an AD‐related target, along with favorable BBB permeability, reinforcing its drug‐like properties (Lawal et al. 2018). The therapeutic relevance of naringenin and related polyphenols is further summarized in Table 3.

**TABLE 3:** Summary of studies showing the anti‐Alzheimer potential of selected polyphenols.

Polyphenol	Mechanism of action	Study findings	References
Resveratrol	Antioxidant, anti‐inflammatory, inhibits Aβ aggregation, promotes Aβ clearance	Reduced Aβ levels and improved cognitive function in AD mouse models.	Capiralla et al. (2012)
Resveratrol	Antioxidant, anti‐inflammatory, inhibits Aβ aggregation, promotes Aβ clearance	Suppressed microglial activation and reduced pro‐inflammatory cytokines in vitro.	Zhang et al. (2021)
Resveratrol	Antioxidant, anti‐inflammatory, inhibits Aβ aggregation, promotes Aβ clearance	Enhanced synaptic plasticity and memory in aged rats.	Granzotto and Zatta (2011)
Curcumin	Antioxidant, anti‐inflammatory, inhibits Aβ and tau aggregation, chelates metal ions	Reduced Aβ plaque burden and improved memory in AD transgenic mice.	Yang et al. (2005)
Curcumin	Antioxidant, anti‐inflammatory, inhibits Aβ and tau aggregation, chelates metal ions	Inhibited tau phosphorylation and fibrillization in cell models.	Ma, Zuo, et al. (2013b)
Curcumin	Antioxidant, anti‐inflammatory, inhibits Aβ and tau aggregation, chelates metal ions	Attenuated oxidative stress and inflammation in AD patients.	Ringman et al. (2012)
Quercetin	Antioxidant, anti‐inflammatory, inhibits Aβ aggregation, enhances mitochondrial function	Reduced Aβ‐induced toxicity and improved neuronal survival in vitro.	Ansari et al. (2009)
Quercetin	Antioxidant, anti‐inflammatory, inhibits Aβ aggregation, enhances mitochondrial function	Decreased oxidative stress and improved cognitive performance in AD mice.	Sabogal‐Guáqueta et al. (2015)
Quercetin	Antioxidant, anti‐inflammatory, inhibits Aβ aggregation, enhances mitochondrial function	Enhanced autophagy and reduced tau pathology in cell models.	Godoy et al. (2017)
Kaempferol	Antioxidant, anti‐inflammatory, inhibits Aβ aggregation, promotes autophagy	Reduced Aβ‐induced neurotoxicity and improved memory in AD mice.	Jafari et al. (2022)
Kaempferol	Antioxidant, anti‐inflammatory, inhibits Aβ aggregation, promotes autophagy	Inhibited tau phosphorylation and aggregation in neuronal cells.	Kim et al. (2018)
Kaempferol	Antioxidant, anti‐inflammatory, inhibits Aβ aggregation, promotes autophagy	Attenuated oxidative stress and inflammation in AD models.	Devi et al. (2015)
Naringenin	Antioxidant, anti‐inflammatory, inhibits Aβ aggregation, enhances synaptic plasticity	Reduced Aβ levels and improved cognitive function in AD mice.	Heo et al. (2004)
Naringenin	Antioxidant, anti‐inflammatory, inhibits Aβ aggregation, enhances synaptic plasticity	Inhibited tau hyperphosphorylation and improved neuronal survival in vitro.	Ghofrani et al. (2015)
Naringenin	Antioxidant, anti‐inflammatory, inhibits Aβ aggregation, enhances synaptic plasticity	Enhanced mitochondrial function and reduced oxidative stress in AD models.	Zbarsky et al. (2005)

## 6 In Silico Support by Molecular Docking and Network Pharmacology Study

The study employed a systematic and multi‐step computational approach to identify and evaluate polyphenols with potential therapeutic effects against AD. Initially, a comprehensive dataset of 651 polyphenols was curated from Dr. Duke's Phytochemical and Ethnobotanical Database (https://phytochem.nal.usda.gov/chemical‐polyphenols) (Duke 1994). After removing 62 duplicates, 589 unique polyphenolic compounds were retained for further analysis. From these, the top 13 molecules were selected based on their strong antioxidant, anti‐inflammatory, and anti‐AD properties, as evidenced by prior research or computational predictions. To elucidate the molecular mechanisms of these polyphenols, their protein targets were identified using the SuperPred database, which provided UniProt IDs, and the STRING database, which facilitated the mapping of target gene names. The GeneCards database was utilized to predict AD‐associated genes, enabling the identification of potential therapeutic targets (https://www.genecards.org/). Pathway analysis was conducted using Cytoscape‐v3.10.3 software to visualize complex interactions and signaling pathways involved in AD pathogenesis.

For molecular docking studies, the three‐dimensional structure of the target protein (PDB ID: 1UV5) was obtained from the Protein Data Bank (https://www.rcsb.org/), a repository maintained by the Research Collaboratory for Structural Bioinformatics (RCSB). This particular PDB entry was selected based on its X‐ray crystallographic origin, favorable resolution (< 2.8 Å), and high percentile scores in global validation metrics, indicating superior structural quality. Prior to docking, the protein structure was preprocessed using PyMOL software (version 2.5) (DeLano 2002) to remove water molecules, add missing hydrogen atoms, and prepare the structure for subsequent analysis. The protein was prepared using the Protein Preparation Wizard in Maestro 12.5, followed by grid generation to define the binding site (Buzdar et al. 2025). Ligand preparation was performed to optimize the polyphenol structures for docking using software Maestro 12.5. Molecular docking was carried out to predict binding affinities and interactions between the polyphenols and the target protein. The docking results were analyzed, and the top‐scoring complexes were visualized in 2D and 3D using Discovery Studio 2024 to interpret binding modes and interactions at the molecular level (Zareei et al. 2024). This integrated approach combined cheminformatics, bioinformatics, and molecular modeling to identify promising polyphenolic candidates for AD therapy, providing a robust framework for further experimental validation.

**FIGURE 3:**
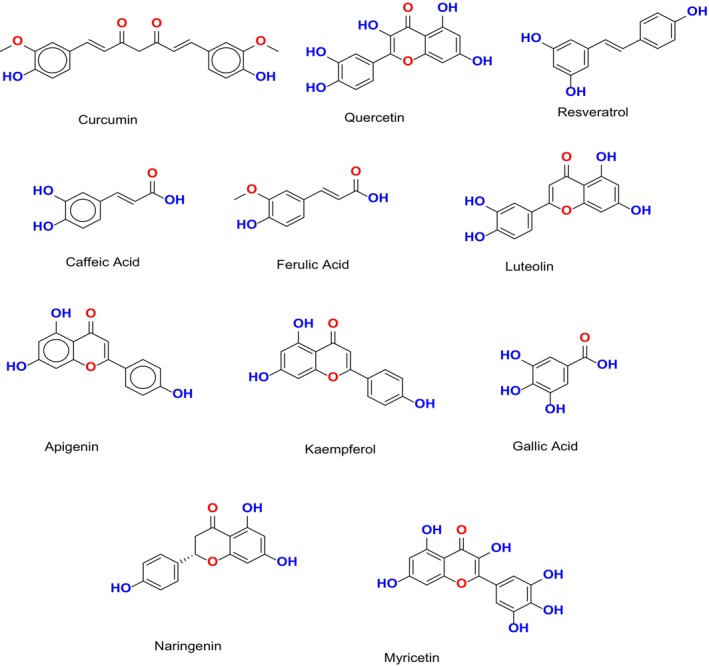
Chemical structures of selected polyphenolic compounds exhibiting neuroprotective potential against Alzheimer's disease (Created by ChemDraw Office).

The polyphenolic compounds depicted in Figure 3 represent a multifaceted approach in combating AD by targeting its core pathological pathways, including oxidative stress, neuroinflammation, Aβ aggregation, and tau pathology. These bioactive molecules exhibit diverse and overlapping mechanisms of action, positioning them as strong candidates for multi‐target drug development. These results supported by (Sharifi‐Rad et al. 2022), who emphasize the broad spectrum efficacy of phytochemicals such as curcumin (80–500 mg/day), resveratrol (150–500 mg/day), quercetin (500–1000 mg/day), and (EGCG; 300–400 mg/day), highlighting their roles in modulating oxidative and inflammatory pathways, as well as Aβ and tau pathology. These compounds exert antioxidative effects by upregulating endogenous antioxidant systems Nrf2/ARE pathway, attenuate neuroinflammation by inhibiting NF‐κB, and interfere with amyloid fibril formation and tau phosphorylation. Building upon this, (Jin‐Feng et al. 2025) provide additional evidence supporting the multi target potential of these polyphenols in preclinical models. In their study, curcumin (100 mg/kg/day), resveratrol (30 mg/kg/day), and EGCG (50 mg/kg/day) were shown to significantly mitigate AD‐related pathology in animal models through a synergistic mechanism involving oxidative stress reduction, neuroinflammatory suppression, and inhibition of β‐amyloid aggregation.

### 6.1 Therapeutic Targets of Polyphenols Against Alzheimer's Disease

**FIGURE 4:**
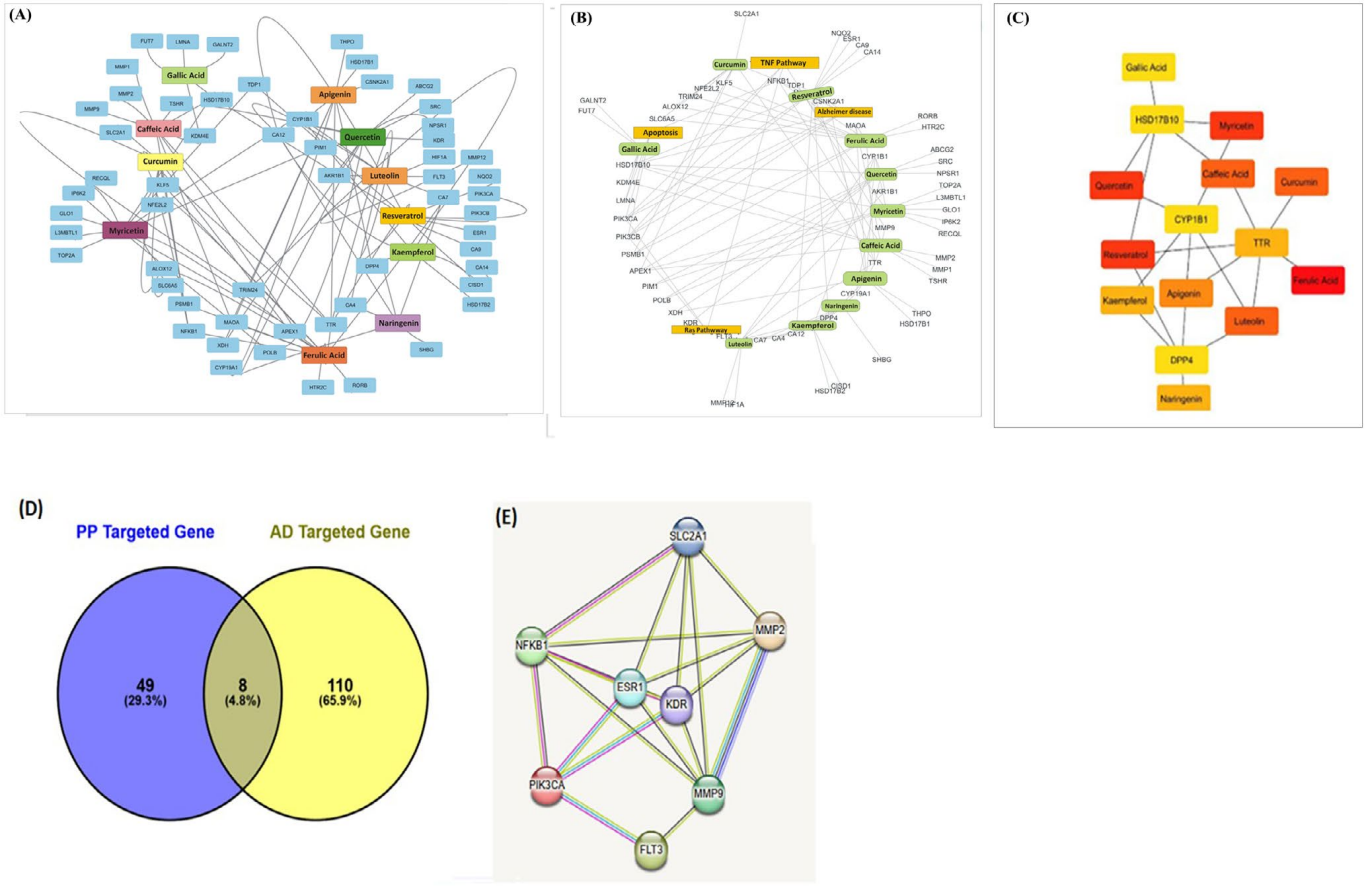
Network analysis of polyphenolic compounds targeting genes relevant to Alzheimer's disease. (A) Network of polyphenolic compounds and their diverse molecular targets like, enzymes, receptors, and signaling pathways. (B) Molecular pathways targeted by polyphenolic compound genes. (C) Shortest targeted pathway of polyphenolic compounds. (D) Venn diagram showing overlap between polyphenol‐targeted genes and Alzheimer's Disease‐Related Genes. (E) Protein–protein interaction network of key targeted pathway components.

The polyphenolic compounds exhibit their therapeutic potential in Alzheimer's disease (AD) by interacting with a diverse array of molecular targets, including enzymes, receptors, and signaling pathways, to modulate oxidative stress, inflammation, and neurodegeneration. For instance, Gallic acid, a phenolic acid with strong antioxidant properties, targets the NFKB1 gene, regulating pathways involved in oxidative stress and inflammation (Thottappillil et al. 2024). Similarly, Caffeic Acid interacts with Xanthine Dehydrogenase (XDH) to mitigate oxidative damage (Iraz et al. 2006), while curcumin, known for its neuroprotective and anti‐inflammatory effects, targets CYP19A1 and NFKB1, influencing estrogen metabolism and inflammatory responses (Azzini et al. 2024). Myricetin inhibits Arachidonate 12‐Lipoxygenase (ALOX12), modulating inflammatory pathways and protein degradation (Jang et al. 2020), whereas Apigenin targets SRC and ESR1, impacting cell signaling and hormone regulation (Zhao, Wang, Wang, et al. 2013). Quercetin interacts with DPP4 and HTR2C, affecting metabolic and neurological processes (Khan et al. 2019; Zaplatic et al. 2019), while Kaempferol and Luteolin target MMP1 and MMP2, involved in inflammation and tissue remodeling (Alexander et al. 2023). Additionally, (Arul et al. 2012) naringenin modulates SHBG and RORB, regulating hormone activity and circadian rhythms (Figure 4A). These interactions highlight the multifaceted mechanisms by which polyphenols exert their therapeutic effects, targeting key pathological processes in AD, including oxidative stress, neuroinflammation, and synaptic dysfunction, thereby offering a promising avenue for therapeutic intervention.

The complex gene interaction network in Figure 4B illustrates the common pathways through which polyphenolic compounds like Myricetin, Luteolin, Kaempferol, Caffeic Acid, Quercetin, Apigenin, Curcumin, and Ferulic Acid exert their therapeutic effects against Alzheimer's disease (AD). These compounds target key genes such as *SLC2A1*, *ESR1*, *NFE2L2*, *NFKB1*, *MAOA*, *CYP1B1*, *AKR1B1*, *MMP9*, *TTR*, *CYP19A1*, *DPP4*, *HSD17B10*, *PIK3CA*, *PIK3CB*, *XDH*, *KDR*, *SRC*, *TOP2A*, and *GLO1*, which are involved in critical AD‐related processes. For instance, *NFE2L2* regulates oxidative stress (Chandran et al. 2023), *MAOA* and *TTR* are linked to neurodegeneration, *NFKB1* modulates inflammation, and *PIK3CA* and *SRC* influence cellular signaling pathways (Long et al. 2021; Sousa et al. 2004). Notably, Curcumin targets *NFKB1* and the TNF pathway to suppress inflammation (Wu et al. 2022), while Quercetin and Myricetin interact with *CYP1B1* and *AKR1B1* to mitigate oxidative stress damage (Wang et al. 2024; Zaplatic et al. 2019). These interactions highlight the ability of polyphenols to simultaneously address multiple pathological mechanisms in AD, including oxidative stress, neuroinflammation, and neurodegeneration, underscoring their potential as multi‐target therapeutic agents for AD.

Figure 4C illustrates the shortest pathway through which polyphenolic compounds Gallic acid, Myricetin, Quercetin, Apigenin, and Kaempferol interact with key molecular targets, Hydroxysteroid 17‐Beta Dehydrogenase 10 (HSD17B10) and Transthyretin (TTR), to exert their therapeutic effects. HSD17B10, critical for mitochondrial function and neurodegeneration (He et al. 2023), and TTR, associated with amyloidosis, are modulated by these compounds to regulate pathways linked to inflammation, oxidative stress, and protein aggregation (Eze 2024; Stefani and Rigacci 2013). By targeting these pathways, the polyphenols mitigate oxidative damage, prevent protein misfolding, and reduce neuroinflammation, offering significant therapeutic benefits for neurodegenerative and metabolic disorders. This streamlined interaction highlights the potential of polyphenols to address multiple pathological mechanisms simultaneously, underscoring their promise as multi‐target agents in combating complex diseases like AD.

Moreover, results show a significant overlap between polyphenolic compounds and Alzheimer's disease (AD) at the molecular level, with 49 genes (29.3%) associated with polyphenols and 110 genes (65.9%) linked to AD, including 8 common genes (4.8%) (Figure 4D). Key genes such as Matrix Metallopeptidase 2 (MMP2), Estrogen Receptor 1 (ESR1), and Solute Carrier Family 2 Member 1 (SLC2A1) were identified as central to protein–protein interactions, regulating pathways critical for neurodegeneration, inflammation, and oxidative stress. MMP2 is implicated in inflammation and tissue remodeling (Rivera et al. 2019), SLC2A1 in glucose transport and neuronal energy metabolism, and ESR1 in neuroprotection and hormone regulation (Tian et al. 2024). These interactions suggest that polyphenolic compounds may collectively target core AD mechanisms, including neuroinflammation, tau phosphorylation, and amyloid‐beta aggregation. The findings highlight the strong potential of polyphenols in mitigating AD progression by modulating these pathways, as illustrated in Figure 4E, providing a promising foundation for further therapeutic development.

### 6.2 Molecular Docking Analysis of Polyphenols With Alzheimer's Disease Targets

**FIGURE 5:**
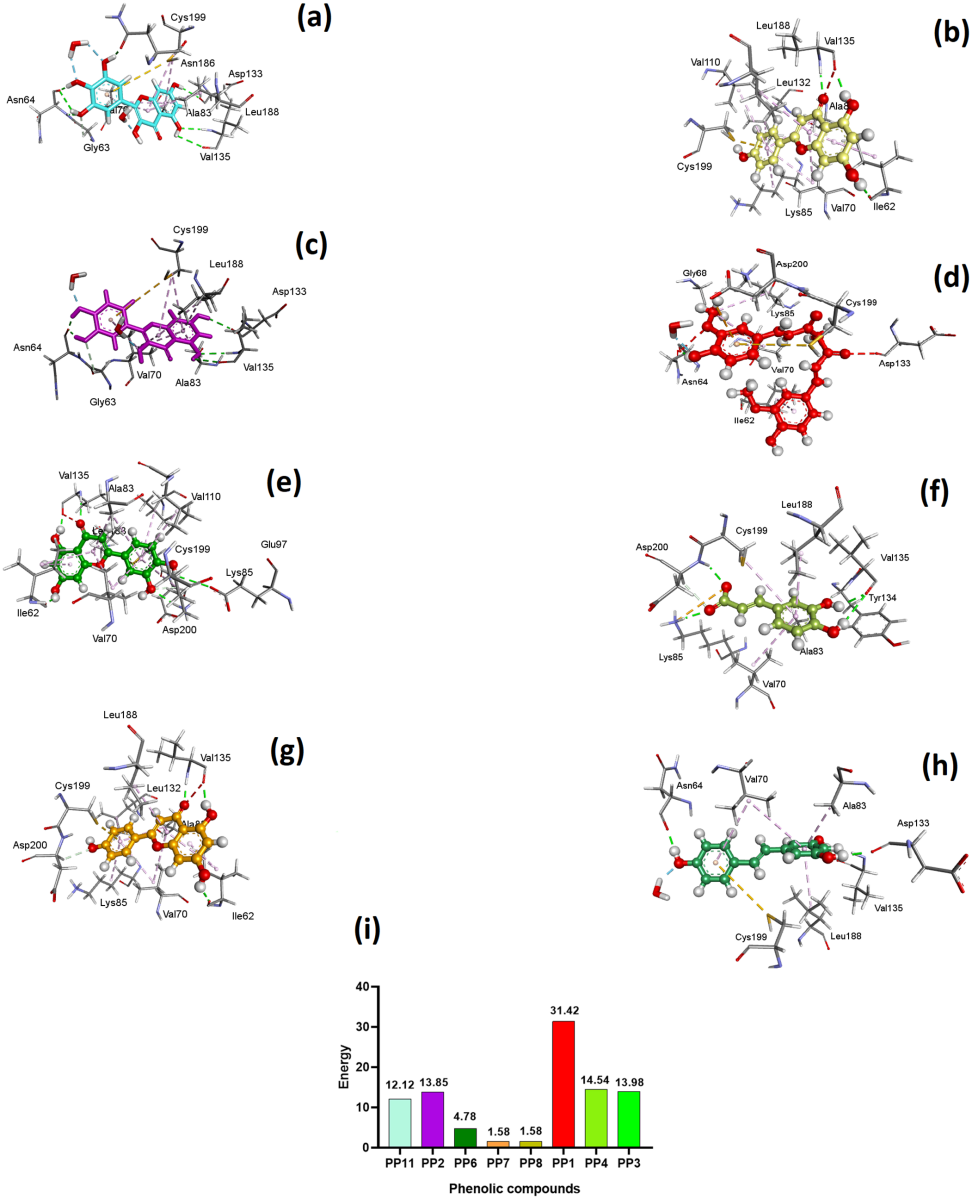
Showing the 3D and 2D interaction of molecular docking analysis of polyphenols with glycogen synthase kinase 3‐beta (GSK3‐β). (a) Myricetin‐GSK3‐β Docking. (b) Luteolin‐GSK3‐β Docking. (c) Kaempferol‐GSK3‐β docking. (d) Caffeic Acid‐GSK3‐β Docking. (e) Quercetin‐GSK3‐β Docking. (f) Apigenin‐GSK3‐β Docking. (g) Curcumin‐GSK3‐β Docking. (h) Ferulic Acid‐GSK3‐β Docking. (i) Docking Scores (kcal/mol).

The molecular docking results demonstrate that polyphenolic compounds, including Myricetin, Luteolin, Kaempferol, Caffeic Acid, Quercetin, Apigenin, Curcumin, and Ferulic Acid, effectively inhibit the ATP‐binding region of glycogen synthase kinase‐3 beta (GSK3‐β), a key enzyme implicated in AD pathology (Zareei et al. 2024). These compounds form specific hydrogen bonds and hydrophobic interactions with critical amino acid residues in the ATP‐binding pocket, such as ASN A:64, VAL A:135, ASP A:133, ILE A:62, LYS A:85, and GLU A:97 (Mou et al. 2020). For instance, Myricetin interacts with ASN A:64, ASN A:186, ASP A:133, and VAL A:135 (Murugan et al. 2024), while Quercetin binds to VAL A:135, ILE A:62, LYS A:85, and GLU A:97 (Rani et al. 2024). These interactions stabilize the inactive conformation of GSK3‐β, preventing its hyperactivity, which is associated with tau hyperphosphorylation and neurofibrillary tangle formation (Figure 5a–h). The consistent involvement of residues like VAL A:135 and ASN A:64 across multiple compounds suggests their critical role in the binding mechanism. These findings highlight the potential of polyphenols as GSK3‐β inhibitors, offering a promising therapeutic strategy to modulate tau pathology and mitigate AD progression (Figure 5a–h). Previously, (Youn et al. 2023) demonstrated that marine derived bioactive compounds target multiple AD related pathways, including AChE inhibition, suppression of amyloid‐beta aggregation, and NF‐κB signaling modulation to overcome the AD related pathology. Notably, dieckol demonstrated strong binding affinity to AChE (binding energy: 9.6 kcal/mol) and β‐secretase (BACE1), suggesting its potential as a lead compound. Furthermore, results demonstrate that Glycogen synthase kinase 3‐beta (GSK3‐β), a key enzyme implicated in AD due to its role in tau protein hyperphosphorylation, can be effectively targeted by polyphenolic compounds. Findings of molecular docking using Maestro software revealed that Myricetin, Luteolin, Kaempferol, Caffeic Acid, Quercetin, Apigenin, Curcumin, and Ferulic Acid exhibit strong binding affinities to GSK3‐β (PDB ID: 1UV5), with docking scores ranging from −11.8 kcal/mol (Myricetin) to −8.67 kcal/mol (Ferulic Acid) (Figure 5i). Myricetin showed the highest docking score, indicating superior binding potential, while Apigenin exhibited the highest energy binding capability. The Glide RMSD values for all compounds were consistent, suggesting stable and reliable docking conformations. These findings highlight the potential of these polyphenols to inhibit GSK3‐β hyperactivity, thereby reducing tau hyperphosphorylation and improving cognitive functions in AD models. This supports their therapeutic potential as natural inhibitors for AD treatment.

## 7 Limitation and Future Research Directions

Despite the well‐documented neuroprotective effects of natural polyphenols, several critical limitations hinder their clinical translation in AD therapy. A major barrier is their poor bioavailability, attributed to rapid metabolism, limited systemic retention, and restricted penetration of the blood–brain barrier, necessitating the development of advanced delivery platforms such as nanoformulations, liposomes, or prodrugs to enhance central nervous system targeting (Iqbal et al. 2021). Additionally, the inter‐individual variability in gut microbiota composition significantly influences polyphenol biotransformation into bioactive metabolites, complicating the prediction of therapeutic outcomes and impeding the standardization of treatments (Cardona et al. 2013). The multifactorial nature of AD also poses a challenge, as polyphenols may not sufficiently address all pathological pathways simultaneously. Another limitation lies in the scarcity of well‐designed, long‐term, randomized controlled trials; current studies are often constrained by small cohorts, short durations, and inconsistent formulations, limiting their reproducibility and translational relevance (Vauzour et al. 2010). To overcome these challenges, future research should integrate computational tools to refine drug development and mechanistic insights. Molecular dynamics (MD) simulations using platforms like GROMACS or AMBER can elucidate the stability and interaction dynamics of polyphenol candidates (curcumin, EGCG, resveratrol) with AD‐related targets such as amyloid‐β, tau protein, and acetylcholinesterase, leveraging crystallographic data from PDB entries (Gheidari et al. 2024). Concurrently, machine learning (ML) algorithms such as deep neural networks and random forest models can predict absorption, distribution, metabolism, and excretion (ADME) profiles, neuroprotective efficacy, and off‐target interactions by mining large‐scale omics and pharmacological datasets (Phatak et al. 2009). Moreover, multi‐center, longitudinal clinical trials employing standardized polyphenol formulations are essential to validate efficacy and safety. Addressing these multifaceted gaps through integrative, interdisciplinary strategies may unlock the full therapeutic potential of polyphenols in combating AD pathogenesis.

## 8 Conclusions

In conclusion, polyphenols exhibit promising neuroprotective potential against Alzheimer's disease through their multifaceted roles in mitigating oxidative stress, neuroinflammation, amyloid‐beta aggregation, and tau hyperphosphorylation. By modulating critical signaling pathways such as NF‐κB, Nrf2/ARE, and PI3K/Akt, polyphenols contribute to neuronal survival, synaptic plasticity, and cognitive preservation. The therapeutic efficacy of key polyphenolic compounds such as curcumin, resveratrol, EGCG, and quercetin has been supported by in vitro, in vivo, and emerging in silico studies that elucidate their target interactions and pharmacokinetics. In silico approaches, including molecular docking and ADMET profiling, offer valuable insights into the bioavailability, blood brain barrier permeability, and binding affinity of polyphenols to pathological targets in AD. However, despite compelling experimental and computational evidence, clinical translation remains limited due to challenges in bioavailability, standardization, and long‐term efficacy. Therefore, integrating polyphenol based interventions with advanced drug delivery systems and precision medicine strategies, guided by in silico modeling, may accelerate their development as adjunctive or preventive therapies for AD. Further interdisciplinary research is warranted to validate their therapeutic promise and bridge the gap between laboratory findings and clinical application.

## Author Contributions

**Guifei Chen:** conceptualization (equal), writing – original draft (equal). **Yan Su:** data curation (equal), methodology (equal), writing – review and editing (equal). **Siyu Chen:** formal analysis (equal), software (equal), writing – review and editing (equal). **Tiandong Lin:** formal analysis (equal), validation (equal), writing – review and editing (equal). **Xueying Lin:** conceptualization (equal), supervision (equal), writing – review and editing (equal).

## Ethics Statement

The authors have nothing to report.

## Consent

The authors have nothing to report.

## Conflicts of Interest

The authors declare no conflicts of interest.

## Data Availability

The authors have nothing to report.
